# Current status of research and gaps in knowledge of geophagic practices in Africa

**DOI:** 10.3389/fnut.2022.1084589

**Published:** 2023-02-20

**Authors:** Theophilus C. Davies

**Affiliations:** Faculty of Natural Sciences, Mangosuthu University of Technology, Umlazi, KwaZulu-Natal, South Africa

**Keywords:** geophagy, aetiology, bioavailability, microbiological infections, Africa

## Abstract

This article synthesises current knowledge and identifies research gaps on the still intriguing aspects of the subject of *geophagy* as practised in Africa. Despite the voluminous research literature that exists on the subject, geophagy in Africa is still a largely misunderstood phenomenon. Although the practice is not confined to any particular age group, race, gender, or geographical region, in Africa it is most commonly recorded among pregnant women and children. Till now, the precise aetiology of geophagy remains obscure; but the practice is thought to have both beneficial effects such as having a role as a nutrient supplement, as well as several demerits. An updated critical review of human geophagy in Africa - with a section on (other) animal geophagy -, highlights several aspects of the practice that need further research. A comprehensive bibliography is assembled, comprising some of the more pertinent and recently published papers (mostly post-dating the year 2005), as well as older seminal works, providing a baseline and robust framework for aiding the search process of Medical Geology researchers and those from allied fields wanting to explore the still poorly understood aspects of geophagy in Africa.

## 1. Introduction

*Geophagy* (or *geophagia*), the habit of eating Earth materials (soil, clay, soft stone, wall scraping, sand, termite mound, anthill, dried-up stream sediment, etc.) is a practice that is common throughout the world, among members of the animal kingdom, including humans.

The term “geophagy” is sometimes used interchangeably with the term *pica.* However, according to Young et al. (1), pica refers to a c*raving* and deliberate ingestion of high amounts of non-food items such as ice, chalk, ash, and soil over significant periods of time. Reid (2) added to this definition: “… the compulsive consumption of otherwise normal food items.” Thus, according to Reid (2) and Huebl et al. (3), geophagy can be considered as a form of pica.

In Africa, geophagy remains a largely misunderstood phenomenon, and terms such as “*confusing,”* “*strange,” “mysterious,” “aberrant,” “puzzling,” “curious,” “odd”* and *“perverted,”* have been used at various times when referring to human geophagy. The use of such words to describe the practice, obviously underlines the extent of such misunderstanding. Abrahams (4) comments that: “This is perhaps understandable for members of a developed urban society that is educated, has ready access to modern pharmaceuticals, and which has increasingly, in both a physical and mental sense, become more remote from soils.”

The clinical effects of geophagy are thought to include both the beneficial and the deleterious. There are archeological, biological, cultural, linguistic, religious, symbolic and other dimensions to the phenomenon; and according to Henry and Cring (5), thorough research on these aspects “… may offer a valid paradigm to better understand this sporadic, puzzling, yet human, behavior,” as well as explain factors related to its prevalence, diversity and distribution [see also, (6)]. The ensuing sections of this article are geared toward the identification of a number of these research gaps, based on syntheses of the more pertinent information we have to date (2022) on various aspects of the subject.

This assemblage of current knowledge on geophagy in Africa, and exposure of the gaps in knowledge on the various aspects of the subject are intended to set up a framework and bibliographic pool of references for Medical Geologists and researchers in allied sciences who wish to explore these gaps in knowledge as contributions toward this rather mysterious phenomenon.

## 2. Historical prevalence, diversity and distribution of geophagy in Africa

Early accounts of geophagy, including its historical prevalence, spatial distribution and possible aetiological explanation are given in several early works, including those of Laufer (7), Cooper (8), Halstead (9), and Hunter (10).

Geophagy is a practice observed worldwide, especially among tribal and traditional rural societies; although, up till recently, evidence of the practice did not exist for Japan or Korea (4). Children and pregnant women in rural cultures across Sub-Saharan Africa, Europe, and South Asia are all known to have been practicing geophagy from antiquity. Indigenous peoples of the Americas are also known to have practiced geophagy. Some aborigines in Australia, eat white clay found mostly in the billabounds of the coastal areas of the North territory for medicinal purposes (11).

The practice can be traced back to ancient times, in the days of the philosophers Aristotle and Hippocrates (12). Geophagia can also be traced back to the 18th century when the Sultan of Turkey was known to have been eating a special clay from the island of Lemnos; which led to the adoption by Europeans of the product as a health food (13).

A number of authors affirm that humans first ate soil in Africa. Root-Bernstein and Root-Bernstein (14), for instance, report that: “The oldest evidence of geophagy practised by humans comes from the prehistoric site at Kalambo Falls on the border between Zambia and Tanzania.” At this site, a calcium-rich white clay was found to exist alongside the bones of *Homo habilis* (the immediate forerunner of *Homo sapiens*).

**FIGURE 1:**
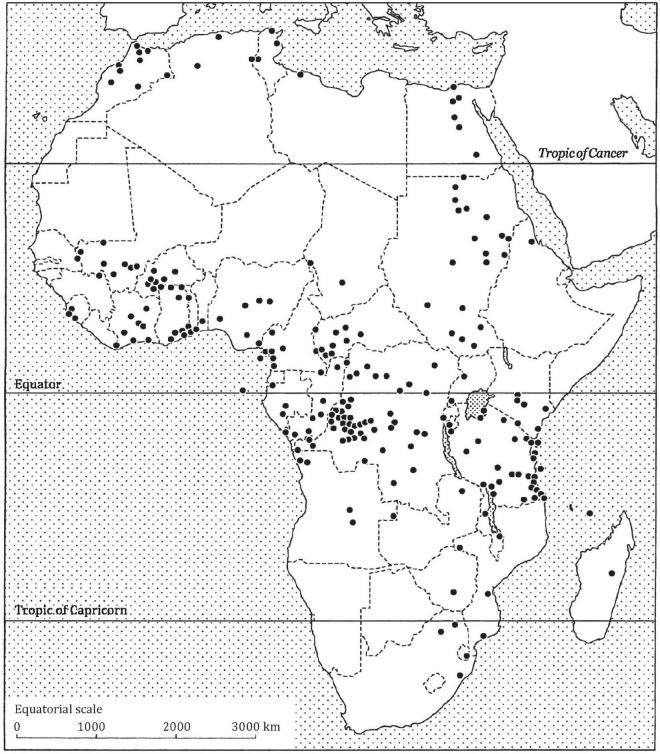
The distribution of geophagy in Africa (shown by the dots) is widespread, though not all countries of the Continent (e.g., some countries in the Sahara) practice the phenomenon. Source: Compiled by Henry and Cring (5) from data by Anell and Lagercrantz (79) and Abrahams and Parsons (131).

Around Africa (Figure 1), this well-established phenomenon is practiced by members of the animal kingdom, including people, especially those from the abounding tribal and traditional rural societies (4). In many other societies outside the Continent, geophagy is generally seen as an unhealthy anomaly [see e.g., (6)]. Among African societies, however, geophagy is generally considered a normally prescribed behaviour. The Medical University of Vienna (MUV) (Medical University of Vienna) (15) gives a figure of between 30 and 80% for the probable number of geophagy practitioners in Africa.

**FIGURE 2:**
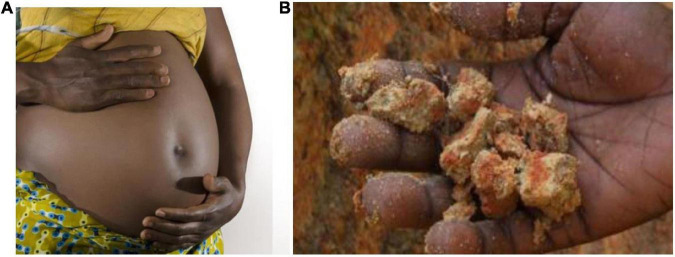
What are the effects of geophagic practices on the developing foetus? **(A)** In sub-Saharan Africa, up to 84% of geophagy practitioners in some regions are thought to be pregnant women. Source: MUV (Medical University of Vienna), (15). **(B)** Geophagic soil consumed by pregnant women on Pemba Island, Zanzibar, a Tanzanian archipelago. Credit: Sera Young, Columbia University Press.

In nearly all societies (worldwide), the highest prevalence of geophagy is recorded among pregnant women [commonly referred to as *geophagy in pregnancy* (GiP) in the literature] and children [e.g., (16)]; but men also engage in the practice, albeit more seldom than women or children. In sub-Saharan Africa, for instance, up to 84 per cent of practitioners in some regions are pregnant women [(3, 17, 18); MUV (Medical University of Vienna), (15, 19, 20); Figures 2A, B]. In Nigeria, the most populous country in Africa, the prevalence of GiP is estimated to be as high as 50% (17). Working in a gold mining area of northwestern Tanzania, (21) reported that 45.6% of pregnant women practiced geophagy, with 54.8% initiating the practice in the first trimester. Njiru et al. (17) study also showed that a total of 101 (65%) of pregnant women ate soil 2 to 3 times per day while 20 (13%) ate soil more than 3 times per day. The amount of Earth material consumed daily varies among geophagists but is typically in the range of between 5 g and 219 g (3).

With such large numbers of geophagy practitioners on the Continent, further research on aspects of this largely misunderstood phenomenon is considered highly justifiable.

In 2013, Abrahams noted that people migrating from societies where geophagy is very common bring about a cultural transfer of the practice to countries that are not typically associated with this deliberate practice. For example, in Britain, geophagy is known to be associated with people who migrated from south Asia (10, 22) and West Africa (23), with the latter (from West Africa) consuming *Calabash chalk* that has been imported from Nigeria and sold in ethnic shops.

Commercial soil samples termed “sikor” originating from Bengal in South Asia is sold in ethnic shops to pregnant Asian women in the UK for practicing geophagists (24, 25). Unfortunately, although there are indications that sikor provides significant quantities of nutritional elements, potential health risks are found to exist for very high iron intake as well as for lead toxicity (24).

**FIGURE 3:**
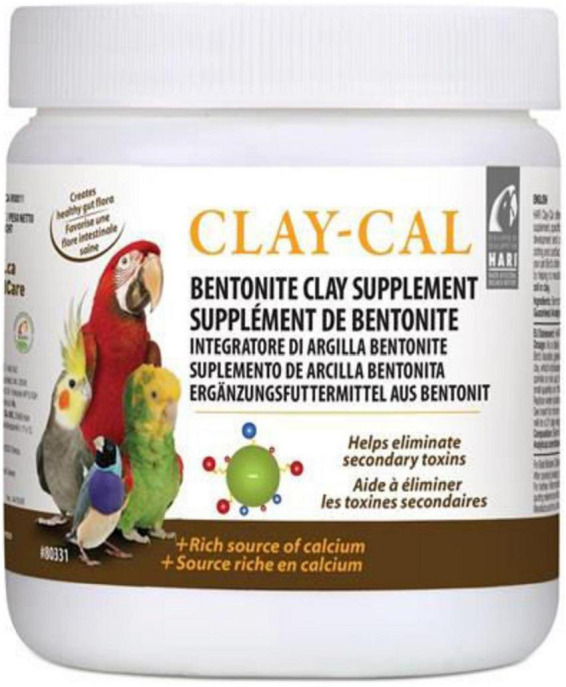
HARI Clay-Cal, a supplement made available by the distributor: La Voliere Parrot Boutique. It is claimed to be an effective calcium supplement for pet birds whose seed diets are low in calcium. Source: http://usa.hagen.com/File/Image/m/275/250/0707dccc-1372-4f2c-8106-6fbe4dae2827 (accessed 09.03.2020).

**FIGURE 4:**
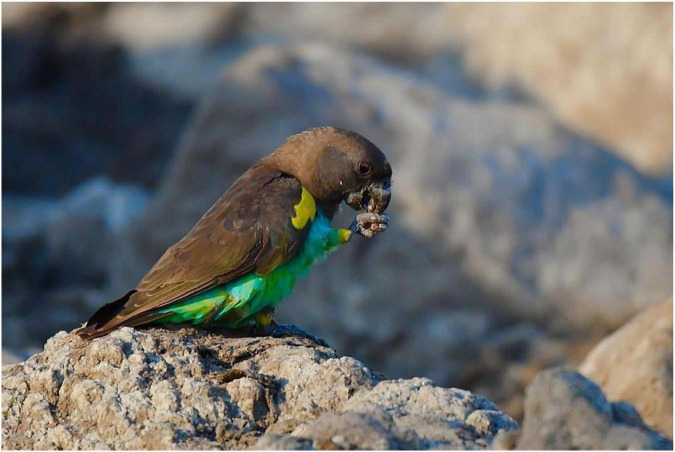
Meyer’s parrot eating clay at a bird hide in Kafue National Park, Zambia. Credit: Butsfons. Source: https://www.flickr.com/photos/cirdan-travels/38709238452 (accessed 29.10.2022).

Similarly, MUV (Medical University of Vienna), (15), noted the habit of eating soil to be prevalent among some migrants from Africa to Europe, in particular, Vienna in Austria, where that particular study was conducted. They buy portions of geophagic material from exotic supermarkets and health food stores that also offer pharmaceutical additives such as bentonite clay for internal use (Figure 3; (4); MUV (Medical University of Vienna), (15); See also Section “5.2. The detoxification hypothesis,” this article).

In researching the distribution of geophagy worldwide, it has to be noted that there is a large degree of underreporting of the phenomenon [see e.g., (1, 3, 21)]. Reasons advanced for underreporting include embarrassment regarding the behaviour, lack of knowledge regarding craving, and sensitive questioning on the part of certain investigators inquiring about geophagy, as well as the differing perceptions, beliefs; and cultural norms associated with the phenomenon (21).

Also, clinicians do not usually ask patients about their craving for Earth materials (3). Pregnant women might not report their geophagic tendencies, because eating soil does not augur with the hygiene concept associated with western medicine and might feel ashamed or fear chastisement from members of their family or medics (3). who fear that the practice would harm them or their developing foetus (1, 3). Again too, many observers in Africa hold the practice as normal during gestation and therefore might not be seen as necessary to mention (3).

## 3. Contemporary practices

### 3.1. The nature of consumed Earth material

The nature of geophagic materials varies markedly with reference to their types, mode of formation, geochemical composition, and so on. The influence of these variables on consumption patterns and health consequences is the subject of numerous studies [e.g., (26); Young et al. (27); (28–33)], which have in turn, significantly influenced the “aetiological debate” (see Section “5. The aetiological debate,” this article).

### 3.2. Soils of Africa

The soils of Africa are diverse. Almost all of the World Reference Base (WRB) Reference Soil Groups are represented. More than 60% of the soil types represent hot, arid or immature soil assemblages (34).

The greater part of the African land surface is covered by sandy soils (22%), shallow stony soils (17%) and young, weakly developed soils (11%), with only a small area of peat soils.

The composition and texture of many of the soil types are a reflection of the local soil-forming factors such as volcanic activity, accumulations of gypsum or silica, waterlogging, etc. (34). African soils generally contain high levels of iron or aluminium oxides imparting a red colour to the soil.

Soils of the tropical rainforests are often quite fertile depending on the high and constant supply of organic matter provided by the natural vegetation. Soils in Africa can be very old, a feature that underlines the marked changes in climate and vegetation.

In many parts of Africa, soils are losing nutrients at a very high rate, much greater than the levels of fertiliser inputs.

There are limited data in the existing literature on the mineralogical and chemical nature of geophagic clays consumed in Africa [see, e.g., (35)]. African red clays are of diverse origin and of varied mineralogical composition. They are residual in nature, having been produced by weathering of surface rocks under conditions of relatively high temperature and rainfall and good drainage. Leaching of bases and silica leaves the soil rich in iron and aluminium. The plasticity of the clay minerals is modified by the high iron oxide content.

### 3.3. Selectivity of Earth material in human geophagy

The consumed Earth material is carefully selected [see (3, 5, 21, 36, 37)], and can be gathered from a variety of local sources including specific soil horizons, riverbanks and swamps, clay pits, anthills, termite mounds and wall scrapings; or can be bought at local markets, from where it can be transported over long distances.

Henry and Cring (5) noted that the selectivity of soil was something special, being done on the basis of appearance, texture and taste. Earlier research by Geissler (36) observed that children of the Luo tribe in western Kenya preferred material for consumption taken from termite mounds and have a particular liking for the dark red clay collected from the inside walls where the material is considered to be purer. Ogomaka (37) writes: “Different types of Earth materials from these sources are consumed, the material typically containing a high content of clay.” Huebl et al. (3), working in northern Uganda noted that the consumed material needs to have special qualities engendering colour, odour, flavour, softness, and plasticity. Nyanza et al., (21) report that in Tanzania, pregnant women commonly eat soil sticks sold in the market (called *pemba* in Kiswahili), soil from walls of houses, termite mounds, and ground soil (*kichuguu*).

### 3.4. Processing of consumed Earth material

The literature gives several methods used by geophagists to prepare Earth material before consumption. Henry and Cring (5) discuss some of these methods.

The processing of some soils may start with cleaning. The material is mostly air dried, but can also be baked, smoked, salted (3, 38), mixed with herbs or water (39) or flavoured with spices such as *black pepper* and *cardamom* (40). Processing of geophagic materials is generally thought to improve their (food) quality in terms of appearance, texture and palatability (26, 41), and freedom from harmful organisms such as helminth ova (38).

There are other specialised techniques for the isolation and purification of soil, such as in the removal of microorganisms) for allopathic applications [see: (42)]; but it is doubtful whether such methods would be available to the typical consumer in the rural African setting.

## 4. Geophagy among animals

Geophagy is widespread in the animal kingdom, both small and large creatures alike consuming some form of Earth material and for some purpose, *which, to date, in many cases, remain unclear*. Galen (130 - 200 CE), the Greek philosopher and physician, was the first to observe how sick or injured animals used clay for healing purposes in the second century AD (43, 44).

The practice has been recorded in all the chordate orders, being particularly common in birds, mammals, reptiles, and fish (5, 45). Some invertebrates such as earthworms and termites also indulge in the practice, but much of the recent research has focussed on mammals, from bats to zebras, and on primates, especially monkeys, macaques, and chimpanzees, as well as a variety of ungulates (46). *There is however, a huge gap still existing in our understanding of different aspects of geophagy in animals, especially the non-human primates* [see Section “8. Suggested areas for further research” (10i)]. *The factors driving the process, for instance are still not yet firmly established* (47, 48). In the case of avian geophagy, some evidence suggests that sodium is the most important driver [see e.g., (49)].

### 4.1. Consumption patterns in animal geophagy

According to Engel (39), it appears that geophagy is far more common in animals that subsist on a diet predominantly consisting of plant food. The presumption is that they eat Earth materials for the purpose of gaining minerals, such as salt (sodium chloride), lime (calcium carbonate), copper, iron, or zinc. The original explanation for geophagy in animals was that although wild animals do seek minerals from natural deposits, a need for minerals could not by any means provide a universally accepted explanation for geophagy (39) in all practitioners in the animal kingdom.

Similarly, in bats, the debate continues over whether geophagy is primarily for nutritional supplementation or for detoxification. Some researchers do believe that certain species of bats regularly visit *mineral- or salt licks* (places where animals can go to *lick* essential *mineral* nutrients from a deposit of *salts* and other minerals) to increase mineral consumption [see e.g., (50)]. However, Voigt et al. (51) demonstrate that both mineral-deficient and healthy bats visit salt licks at the same rate. *In the absence of incontrovertible scientific evidence to date, therefore, mineral supplementation is unlikely to be the primary reason for geophagy in bats.*

Parrots are known to eat toxic foods globally, but geophagy is concentrated in very specific regions [(52); See Section “4.2. Selectivity of Earth material in animal geophagy,” this article]. Lee et al. (52) further showed that parrots in South America practice their geophagy at sites with a significantly positively correlation with distance from the ocean. This correlation can be interpreted as the parrot’s preference for particular geophagic sites being based on an overall lack of sodium in the ecosystem, rather than variation in food toxicity, in accounting for the spatial distribution of geophagy.

Also, it has been observed that presence at salt licks increases during periods of high energy demand [see e.g., (53)]. This is a feature especially evident in lactating and pregnant bats, as their food intake increases to meet higher energy demands. Voigt et al. (51) concluded that “… the primary purpose for bat presence at salt licks is for detoxification purposes, compensating for the increased consumption of toxic fruit and seeds.”

### 4.2. Selectivity of Earth material in animal geophagy

As is the case with geophagy in humans, the type of Earth material consumed by animals is carefully selected. Regular visitation of salt licks has been reported among African forest elephants (54), besides many other animals engaged in geophagy. Wild birds show that they often prefer clayey soils and clayey sediments consisting largely of minerals of the smectite family of clay minerals that includes montmorillonite and bentonite (49).

The preference for certain types of clay or soil can lead to peculiar feeding behaviour. In Africa, avian species showing geophagy can be broadly divided into those congregating and feeding on grit, and those feeding on clay (55–57). Parrots, for instance, avoid eating the substrate in layers one metre above or below the preferred layer [(56, 57); Figure 4].

The preferred soil bands are shown to have much higher levels of sodium than those that are not chosen.

## 5. The aetiological debate

Up until now, many theories have been advanced to account for the aetiology of Earth material consumption, many of which are still largely unsubstantiated. The intensity of this long-standing debate on causative factors is still gathering pace and will only gradually diminish as present-day researchers continue to focus their effort on the remaining knowledge gaps, a number of which are tabulated under Section “8. Suggested areas for further research.”

The abounding theories about why people practice geophagy are legion. These include: as a nutritional supplement; as food detoxifier; as diarrhoeal pharmaceutical; soothing gastrointestinal or gastroaesophageal reflux disorders such as hypersalivation, heartburn, spitting and vomiting during pregnancy; as famine food; as a natural stimulant; boosting apetite; for psychological (comforting reasons), neuropsychiatric, cultural and religious reasons, and many more [see: (3, 6, 7, 17, 32, 58, 59) and others]. The symbolic dimension of geophagy cannot be overlooked. Henry and Cring (5) describe “… how eating soils can mean more than simply fulfilling a need or a craving” and give evidence of how the mode of practice varies according to social class, sex, and age.

Knudsen (60) observes that, for the Chaggas of Tanzania, geophagy appears to be sacred to women, and, according to Woywodt and Kiss (61), South African urban women, ingest soils for enhancement of their beauty.

### 5.1. The micronutrient supplementation theory

Some of the foregoing theories and causal explanations seem particularly robust, especially those that centre around geophagy’s medicinal efficacy, which is predicated on the idea that geophagy, [in the words of Engberg (6)] “… is a rational behaviour for people living in environments and social situations that do not otherwise adequately accommodate their vitamin and mineral requirements.” This thesis gathers strength, especially when considered in the context of pregnancy [e.g., (17)], in which case, many practitioners believe that the soil or clay affords nutrients and minerals, such as iron, calcium, and potassium or vitamins such as cobalamin, that may otherwise be absent from the diet, but whose transfer to the developing foetus is quintessential for its optimal development.

#### 5.1.1. How far established is the micronutrient supplementation theory as a causal factor for geophagy?

Present day researchers on the subject of geophagy generally agree that one of the principal values is the curbing of micronutrient deficiencies, especially in GiP; and this remains the most pervasive and perhaps the most credulent explanation of human geophagy so far [see e.g., (17, 59, 62, 63)].

**FIGURE 5:**
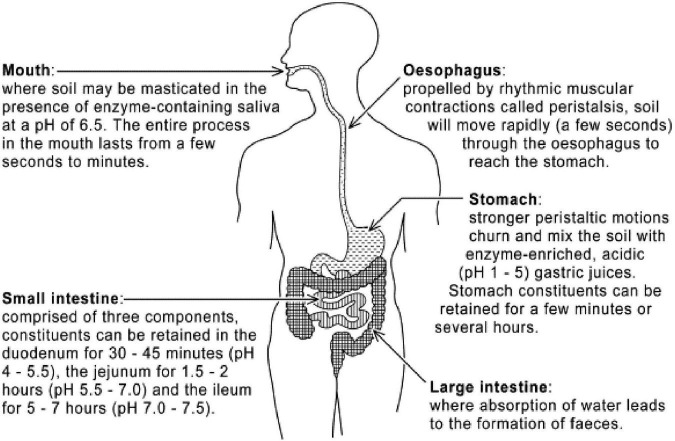
The human digestion system: Conversion of soil into absorbable substances in the gastrointestinal tract starts in the mouth and continues in the stomach and intestines. Source: Abrahams (91).

The strength of the nutrient supplementation theory (Table 1) is predicated on the importance of the direct soil-animal pathway of mineral nutrients that complements the soil-plant-animal route in agricultural systems; and the fact that soils do have the potential to supply mineral nutrients especially iron, and vitamins such as cobalamin where the ingestion of soil (Figure 5) can account for a major proportion of the recommended daily intake.

**TABLE 1:** Major hypotheses (micronutrient supplementation and detoxification attribution) on geophagy.

Sources	Nutrient hypotheses	Detoxification hypotheses
Abrahams and Parsons (131)	Minerals - especially iron	Detoxification (bimodal)
Brevik (141)	Enhanced mineral nutrition	–
Hooda and Henry (143)	Calcium deficiency	Detoxification
Johns and Duquette (146)	Calcium, sodium	Acorn tannins
Johns (145)	–	Detoxification
Vermeer (152)	Calcium deficiency	Plant toxins
Young (155)	Calcium deficiency	Detoxification
Wiley and Katz (154)	Calcium and other minerals	Detoxification (bimodal)

Source, from: Henry and Cring (5): Table 8.1.

The consumption of soil for supplementation of iron and calcium would depend on the concentrations of these elements in the soil, which, to a large extent depends on the type of soil and the degree of weathering the soil has undergone (64–66); chemical weathering, being one of the main processes by which weathered material is altered prior to deposition.

#### 5.1.2. Cobalamin supplementation

According to Rosenthal et al. (67), women of childbearing age from low-resource settings and those with low intake of animal products are the ones often at risk of cobalamin (vitamin B12) deficiency. An increased store of cobalamin being quintessential during pregnancy and lactation to meet the demands of the mother, the foetus, and the infant (68–70).

Humans cannot synthesise cobalamin [e.g., see (68, 71)]; and the only way it is obtained is through dietary intake (72). Geophagy may therefore be a behavioural adaptation to obtain cobalamin produced by bacteria and archaea in the soil. *More research on the role of geophagy in supplementing cobalamin (and possibly inducing pica) in pregnancy is warranted (see section “8. Suggested areas for further research,” this article).*

### 5.2. The detoxification hypothesis

The microbiological effects of clay consumption to animal health have been known for a long time [see e.g., (73)]. These effects include binding of mycotoxins (fungal toxins), bacterial endotoxins (internal toxins), manmade toxic chemicals, parasites, and pathogens.

Detoxification of harmful substances present in the diet of individuals by soils, and the relief from gastrointestinal disorders depend on the soil sorption capacity, which is determined by its cation exchange capacity (CEC), underlining the necessity of understanding the mineralogy and geochemistry of geophagic soils (see under Section “8. Suggested areas for further research,” this article).

Unbaked soil, which is commonly consumed in northern Uganda, for instance, may be microbially contaminated and cause gastrointestinal upsets (3). Clay protects the gut lining from corrosion, acts as an antacid and curbs diarrhoea.

According to Kreulen (74), addition of bentonite clay (Figure 3) which is sold worldwide as a digestive aid, can improve food intake, feed conversion efficiency, and absorption patterns in domestic cattle by 10–20%. Veterinarians therefore find bentonite clay an effective antacid that can bring relief to clay-fed cattle having some form of gastrointestinal malaise (39).

The effectiveness of bentonite clay as an antacid derives from its special properties (hydration, swelling, water absorption, viscosity and thixotropy), making it a valuable material not only as the base for some medicines, but also for several other uses and applications.

Similarly, kaolin [mainly comprising the clay mineral kaolinite (Al_2_O_3_(SiO_2_)2(H_2_O)_2_)], is widely used as a digestive aid and is the base for some medicines, such as Kaopectate, for suppressing diarrhoea and reducing toxic effects and inflammation in the digestive system.

Attapulgite (sepiolite and palygorskite), another type of clay, is structurally different from bentonite and kaolin, and is an active ingredient in many anti-diarrhoeal medicines [see, e.g., (75)].

## 6. Social, cultural, psychological and religious perspectives on geophagy

The association between geophagy and spiritual and religious beliefs, commented on by Frate (76) way back in 1984, has also more recently been revisited [see e.g., (6, 36); MUV (Medical University of Vienna), (15)]. In 2012, Brevik and Burgess noted geophagy’s early historic relationship with religion, which “… ranges from the use of antique lozenges of *terra sigilatta*, extracted by a priestess and mixed with goat’s blood, to the clay tablets marked with Roman Catholic symbols and images of the cult of Esquipulas in Guatemala.” Consumption of Earth material from sacred sites for its expected healing properties has also been noted in India (7) and New Mexico (58).

In Africa, the eating of soil has come to be seen as a socially accepted practice in many quarters, and a common habit in pregnant women. In studying geophagy as practised among the Luo community of western Kenya, Geissler (36), describes “… how the practice is associated with social and cultural motives, related to position in the family and community on the one hand and aspects of the meaning of life and one’s place in the world, on the other.”

Geissler (36) further describes how: “… beyond the significance of earth−eating in relation to age, gender and power, it [*geophagy practice*] relates to several larger cultural themes, namely fertility, belonging to a place, and the continuity of the lineage. Earth symbolises female, life−bringing forces.”

The psychological hypothesis centres around the craving ideas wrought by feelings of misery, homesickness, depression and alienation (77).

Other notable recent references on knowledge of social, cultural, psychological and religious perspectives on geophagy as practiced in Africa include those given in Table 2.

**TABLE 2:** Social, cultural, psychological and religious perspectives on geophagy in Africa.

Perspective	Region or country	References
Psychiatric disease	Urban South Africa	Woywodt and Kiss (61)
Religious and cultural underpinnings	Nigeria	Njiru et al. (17)
Religious geophagy	Around the world in Christianity, Islam, Hinduism and hoodoo folk magic	Young (156); Young (157)
Dirt consumption normal in some African cultures	Africa	Henry and Cring (5)
Cultural norm	Africa, India and worldwide	Bhatia and Kaur (140); Prince (151); Vermeer and Frate (153)
Social and cultural factors	Geita District, Tanzania.	Nyanza et al. (21)
Benefits of clay include nutritional, ethnomedical, economic, emotional, aesthetic, and spiritual	The Balengou of the Western region of Cameroon	Pemunta (150)
Cultural factors underlining sociological drivers and cultural evolution of earth eating traditions	Oyi, Anambra State, Nigeria	Okereke et al. (149)
Factors ranging from cultural to religious, inter alia	Imo State, Nigeria	Ogomaka (37)
Cultural motivation, inter alia	Antenatal clinic in Pretoria, South Africa	Macheka et al. (148)
Cultural motivations	African countries: Kenya, Ghana, Rwanda, Nigeria, Tanzania, and South Africa.	Kambunga et al. (20)
Pica, strongly embedded in cultural practice among mothers	Communities around Lake Victoria, Kenya	Izugbara (144); Chung et al. (142)

## 7. Demerits of geophagy

### 7.1. Ingestion of potentially harmful elements

Despite the potential to supply micronutrients, there are a number of apparent risks associated with the practice of geophagy. Earlier studies have suggested that the nutrient value of the soil is overestimated [e.g., EVM (Expert Group on Vitamins and Minerals), (65, 78)], and, contrary to the micronutrient supplementation theory, some researchers [e.g., (66, 79) and (80)] believe that excessive consumption of Earth materials can interfere with bioavailability of micronutrients. Such interference, according to a number of researchers [e.g., (21, 32, 81–86)] can lead to-, or exacerbate, micronutrient and vitamin deficiencies that could cause infectious disease, lead poisoning, bowel impaction, and so on. These conditions can put pregnant woman and the developing foetus at risk.

After initial contact with digestive fluids, micronutrient elements can be solubilised from soils and this *bioaccessible soil content* made available for absorption (Figure 5). However, concern has often been raised about the high concentration levels of some of these elements, those generally referred to as potentially harmful elements (PHEs) in some consumed Earth materials [e.g., (23)]. Although these *total* concentrations may be significantly higher than World Health Organisation guideline limits [see (87, 88)], it is important to take account of the *bioavailability* [defined as the fraction that reaches the human systemic circulation from the gastrointestinal (GI) tract] of soil-PHE (Figure 5). The bioavailability of this PHE is strongly dependant on bioaccessibility, since if an element is not bioaccessible it will not be available for absorption (89), and both bioavailability and bioaccessibility are influenced by a number of soil variables (mineralogy, particle size and morphology) as well as factors associated with the human individual, such as age, sex, genetics and socioeconomic status [WHO (World Health Organisation), (90, 91)].

A number of research groups [e.g., USEPA (United States Environmental Protection Agency), (92–94)] have, in the last two decades, been working on the use of *in vitro* bioaccessibility (IVBA) tests that mimic the conditions of the human GI environment and determine the bioaccessibility of ingested soil chemical elements. However, several problems are evident with the use of these IVBA procedures. For instance, there have (until recently) been a lack of Certified Reference Materials (CRMs) needed for the evaluation of the accuracy of these analyses, and a frequent lack of sufficient *in vivo* information against which the bioaccessible concentrations can be compared.

Despite advances made in the development of these IVBA procedures, there has been only a limited application in understanding uptake dynamics of geophagic materials, largely due to aforementioned criticisms regarding the efficacy of the experimental techniques applied [see (62, 95)].

Ingestion (as in geophagy) of radioactive materials derived from mining of gold, uranium or other minerals associated with ionising radiation emitting substances can occur in localities exemplified by the tin-mining areas of the Jos Plateau in Nigeria [see, e.g., (96)] and the well-known gold and uranium mining sectors of Gauteng Province in South Africa. *This is one gap that urgently awaits further research.*

### 7.2. Microbiological infections

Soil-transmitted helminth infections are caused by different species of parasitic worms. They are transmitted by eggs present in human faeces, which contaminate the soil in areas where sanitation is poor. Approximately 1.5 billion people are infected with soil-transmitted helminths worldwide.

**FIGURE 6:**
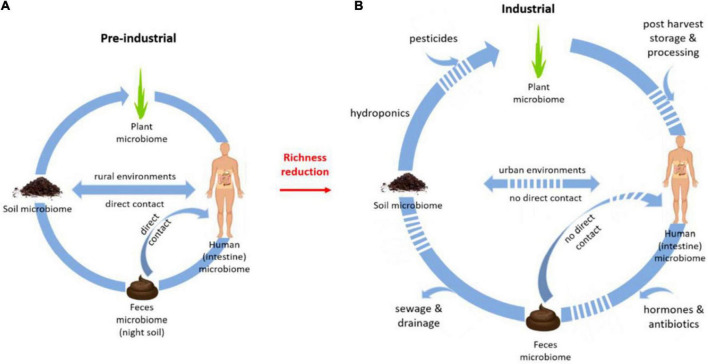
Illustration of how the microbiota in our environment can influence the human intestine microbiome, through direct contact with soil and faeces as well as through food (quality). **(A)** A cycle for pre-industrial microbiota. **(B)** A cycle for industrial microbiota. Source: From Blum et al. (98).

External elements such as helminth ova and faeces may sometimes be present in surface soils in areas where improper biological waste disposal facilities are located; and when consumed, can lead to helminth infections and diarrhoea through interaction with the human intestinal biome [(97–99); Figure 6].

Soil contaminated by industrial pollutants or by human excreta pose considerable threat to geophagists, including infections from various pathogenic soil organisms (100, 101). Helminth infection associated with geophagy has been linked with the frequency of inflammatory bowel diseases (58) as well as an important unrecognised risk factor for environmental enteropathy and stunting (102).

A number of studies [e.g., (101, 103, 104)] have shown that ingested soil is of particular concern as a risk factor for geohelminth infection among children in Africa. However, the species involved, their epidemiology and the kinds of infection they produce remain unclear (105), except in certain cases. Callahan (58) noted that among children in Nigeria, the most common parasitic infection associated with eating dirt is *ascariasis*. Infected children are nutritionally and physically impaired. Control is based on periodical deworming to eliminate infecting worms, health education to prevent re-infection, and improved sanitation to reduce soil contamination with infective eggs. Thankfully, though, safe and effective medicines are now available to control infection.

In adults, geophagy is thought to be an unlikely cause for adult infestation (95, 106), though Ozumba and Ozumba (105) had earlier noted that Hookworm, Ascaris, Trichuris and Strongyloides were common helminths in a mixed-population sample [adults (18 years +), adolescents and children)] in Enugu State of Nigeria. We also now know that helminth eggs, such as Ascaris, which can stay viable in the soil for years, can lead to helminth infections [(107); UCPJ (University of Chicago Press Journals), (108)], even in adults [see, e.g., (109)].

*However, accurate knowledge of the inherent biological dangers of soils contaminated with untreated human or animal waste, has until now, proved difficult to assess; but, through well designed microbiological investigations, it is possible to successfully address this aspect of the problem.*

### 7.3. Other banes of geophagy

There exist a number of other demerits of the geophagic practice in Africa that have been given very little attention in research circles [see e.g., (110, 111)]. Geophagy’s possible association with lead poisoning, blockage of the large intestines, hyperkalemia, phosphorous intoxication and dental injury (112), have been little researched.

Abrahams et al. (24) call for attention to the risk of soil-lead (Pb) toxicity affecting pregnant women and their foetus; whereas, excessive tooth wear and dental enamel damage as consequence of human geophagy were very recently commented on by Ekosse et al. (32). Ingesting soils high in coarse particles could no doubt affect dental enamel (111, 113), and provoke obstruction and rupturing of the Sigmoid Colon (39, 114, 115). *However, the physiological intricacies of these processes remain largely unclear.*

*As of 2016, hardly any research data on appendicitis caused by geophagy existed in the literature* (3). *Tetanus, peritonitis and eclampsia and iron-deficiency anaemia are also considered to pose a further risk* [UCPJ (University of Chicago Press Journals), (108, 116)], *but very few detailed studies on these aspects exist in the contemporary literature.*

*Some attention has recently been drawn on the internal accumulation of soil that can lead to constipation* (101), *intestinal occlusion* (95), *the reduction of the power of absorption of food materials by the body* (117) *and severe abdominal pain* (4), *but more data on these aspects are required for a holistic appraisal of these processes.*

## 8. Suggested areas for further research

Despite the large volume of recently recorded research on the subject of geophagy in Africa, there still exist substantial knowledge gaps in certain aspects of a phenomenon that is still considered largely misunderstood. Some of the more important areas (with potentially high societal impact) of needed research include:

1. *Accurate determination of bioavailability and bioaccessibility of PHEs in consumed Earth material from a particular contaminative source.* This is deemed necessary, given that precise knowledge of these characteristics is critical for site-specific risk assessments.

Despite the progress made in the development of *in vitro* bioaccessibility (IVBA) tests that mimic the conditions of the human GI environment, and determination of the bioaccessibility of ingested soil chemical elements, some methodological constraints still remain [see e.g., (23, 118, 119)]. Refinements in the methodology of systematic *in vivo* studies are therefore recommended on geophagic soils (120). There is also the need for more validation studies in which *in vivo* results are compared with *in vitro* results [see e.g., (121)].

2 (i). *During pregnancy, excessive amounts of soil are often consumed and may have an implication on health, both for pregnant women and for the infant.* Despite its association with anaemia, pregnancy and micronutrients, many ante-natal clinics (ANC) or national guidelines on micronutrient deficiency control are silent on the subject of GiP. The guidelines generally recommend iron supplementation and deworming of pregnant women as anaemia control measures. However, not all women seek antenatal services; hence, *there is need for more innovative ways of addressing micronutrient deficiencies in pregnancy* [see e.g., (17)].

There is therefore an urgent need for intensive and extensive health education regarding the detrimental consequences of this common practice and health promotion in the community. Although geophagy can be part of the topics of “nutrition” and “native medicine” in health education protocols at ANC in many African countries, no local or national guidelines or uniform recommendations on geophagy exist.

2 (ii) The role of geophagy in cobalamin (vitamin B) supplementation during pregnancy and lactation has received little attention [see, e.g., (68)]. The intake requirements for pregnant and lactating women as well as in children need to be re-evaluated in the light of their desperation to regularly consume soil [see e.g., EVM (Expert Group on Vitamins and Minerals), (78)].

2 (iii) The enhancement of intestinal triacylglycerol hydrolysis and non-esterified fatty acid absorption by clay ingestion [see e.g., (122)] as a cause of *pica* among pregnant women needs further investigation.

3. *Examination of geophagic materials for presence of intestinal parasites and their effect on consumers (especially children) of Earth materials* [see e.g., (105, 123)] is highly desirable.

4. *Despite a long period of residence in western countries, geophagy is still a current practice among a significant group of western travellers, who are poorly informed of its harmful effects* [see e.g., (22)]. Therefore, specific information about the risks of geophagy should be transmitted in western countries, and the international importance of geophagy brought out in migration studies and global public health protocols. Preventive education should be integrated into care of HIV adults, not only in Africa, but also in countries outside the Continent into which this category of geophagy practitioners (HIV-infected) have migrated [see (124)].

5. *A structural analysis of the distribution channels of consumed Earth materials should be determined by identifying the participants and their relationships (i.e., equal, collaborative, exploitative).* It is also necessary to establish the extent to which stakeholders in the distribution chain participate in the exploitation, preparation and marketing of the soil products.

6. *Soil mineralogy also plays an important role in the behaviour of soils, along with the soil texture and organic matter content* (32, 125), *and contributes to the effect of geophagy among humans through its influence on soil cation exchange capacity (CEC)* (126). For these reasons, it is important for researchers studying the direct soil-animal pathway of mineral nutrients to have a thorough understanding of the mineralogy, geochemistry and source characterisation of geophagic materials. A lot of progress has been made on these kinds of studies [e.g., (20, 32, 127–130)], but much more needs to be done.

7. *The association between human geophagy and age, gender and educational status appears to be still conjectural* [see e.g., Engberg (6), Abrahams and Parsons (131); Geissler (36), Brevik and Burgess (54), Golden et al. (132)]. In the animal kingdom, the association between geophagy and age and gender appears to be clearer [see e.g., Holdø et al. (47), Pebsworth et al. (133), Young et al. (1), Myers (134)]. More research on these associations needs to be undertaken.

8. *The role of clay minerals (e.g., bentonite clay, attapulgite) as a base for certain pharmaceuticals (digestive aid; anti-diarrhoeal medicines) should be further researched.* As Moosavi (135) writes: “As traditional remedies seem to have a deep root in maintaining body health, it merits doing more research works on bentonite clay and its impacts on body function.”

9. Given that the causative reasons for male geophagy appear to differ from those of female geophagists [(e.g., “Many men believed that eating clay increased sexual prowess, and some females claimed that eating clay helped pregnant women to have an easy delivery.” (136)*], geophagy in men should be included in further studies and should form part of every health education protocol.*

10 (i). *The role of geophagy among non-human primates is still far from well understood.*

More data are required on the behavioural and dietary characteristics, as well as preferential soil types consumed, in order to more rigorously investigate the hypotheses of *protection* and *mineral supplementation* across representative species of all taxonomic groups, geographical regions, and dietary classification [see e.g., (137)].

10 (ii). *The role of geophagy in species conservation and biodiversity for many endangered species in Africa needs further attention. For example, despite baboons’ widespread distribution across Africa, geophagy among all subspecies of these primates has been poorly documented* [see e.g., (133)].

Also, the adaptive functions of geophagy, found in a number of bird species in Africa remain unclear [see e.g., (55)]. Indeed, as recently as 2014, Lee and Marsden (147) wrote: “… *we still do not know how common and widespread geophagy is in birds - largely because it is difficult to observe in the vast and little-known tropical forests.”* Factors driving avian geophagy is therefore another area awaiting further investigation [see e.g., (49, 52)].

11. *Finally, we need to answer the question of whether humans can practice geophagy safely, in cases where the practice cannot be discontinued (refer to the merits of processing the material before consumption, such as drying, baking, smoking and salting it).* In urban areas in particular, health education (138) should also explain where unpolluted soils can be found, and which areas should be avoided.

## 9. Value-added contributions made

This article provides an updated critical review of the practice of geophagy in humans and other animals in Africa, and outlines theories about the causal factors of the practice in different groups. It provides a sound basis and robust framework for exploring the several gaps in knowledge that are awaiting research. These are imperatives that represent the crucial roles served by systematic reviews and are in accordance with the “Preferred Reporting Items for Systematic Reviews and Meta-Analyses” (PRISMA) statement of 2020 [see: (139)].

The overview presented, it is hoped, would lead to the evolution of a more unified approach in the design of *in vitro* studies in bioavailability and bioaccessibility studies in geophagy. The several gaps in knowledge revealed, and the comprehensive list of recent references given, would underpin the search process of researchers wanting to explore the subject further, and expedite the formulation of tangible proposals of remedial interventions for (reducing) harmful effects or deriving optimum benefits from the practice of geophagy in Africa.

## 10. Conclusion

The aetiology of geophagy remains elusive. Both physiological (e.g., mineral deficiency or hunger) and psychological (e.g., craving, obsessive-compulsive spectrum disorder) models have been proposed. Cultural and socioeconomic factors have also been identified as influencing the practice of geophagy, thereby highlighting its complex and little understood nature.

The health impacts of geophagy also remain controversial and inconclusive, with reports in the literature showing the practice to have both health benefits and harmful effects; as well the absence of effects (a. Substances with clay constituents have long been used (e.g., Kaopectate^®^) for treating gastroenteritis, nausea, diarrhoea and vomiting.

Among the demerits of the practice of geophagy, a major concern, especially among children, is helminth infection, acquired through ingestion of soil contaminated with faecal matter. This can lead to anaemia due to blood loss from the intestine.

Research on various aspects of geophagy, once conducted in separate, respective, fields and shaped by different paradigms, is now generally pluridisciplinary, an approach predicated on the evolution of our current understanding (non-understanding) of the phenomenon.

In conclusion, highlighted in this paper are the biological, physical, cultural, religious, symbolic and other dimensions of geophagy. Several findings have emerged about the basis and effects of the behaviour and about the research conducted till now, to shed light on the many aspects that are still unclear or unknown. The avenues for further research on geophagy are legion and a number of them are listed under Section “8. Suggested areas for further research,” this article.”

## Author contributions

The author confirms being the sole contributor of this work and has approved it for publication.
